# Spontaneous Remission of Acute Lymphoblastic Leukemia Following Candida tropicalis Fungemia

**DOI:** 10.7759/cureus.62435

**Published:** 2024-06-15

**Authors:** Benjamin J McCormick, Hamayun Imran

**Affiliations:** 1 Internal Medicine, Mayo Clinic, Jacksonville, USA; 2 Pediatric Hematology/Oncology, University of South Alabama, Mobile, USA

**Keywords:** acute lymphoblastic leukemia (all), candidemia, fungemia, candida tropicalis, pediatric cancer, acute leukemia, spontaneous remission

## Abstract

Spontaneous remission (SR) in acute lymphoblastic leukemia (ALL) is a poorly understood phenomenon that has been sporadically reported in medical literature for over a century, and the molecular and immunologic mechanisms of remission pose interesting clinical questions. Furthermore, the often-transient nature of these remissions poses a challenge to physicians in formulating an approach to treatment. We report on a rare case of *Candida tropicalis* sepsis in a three-year-old female with high-risk ALL who received less than two months of treatment prior to sepsis and subsequent SR.

## Introduction

Spontaneous remission (SR) in high-grade cancer is a poorly understood phenomenon that has been documented sporadically throughout medical literature [1-4]. SR can be defined as the disappearance of a malignant neoplasm following either treatment that is considered insufficient to cause a significant change in malignant disease or the absence of treatment altogether [1]. In this report, we present a case of high-grade acute pre-B acute lymphoblastic leukemia (ALL) in a child whose treatment was complicated by a systemic *Candida tropicalis* infection followed by transient CR of ALL. To the best of our knowledge, this is the first reported case of SR following *Candida tropicalis* sepsis in pediatric high-risk ALL.

## Case presentation

A three-year-old Caucasian female presented with a five-day history of subjective fever, oral stomatitis, unilateral leg pain, and pain in the vaginal area. On the day of admission, she experienced a bout of severe hematemesis. On initial exam in an outpatient setting, she exhibited hepatomegaly and mild cervical lymphadenopathy, and the working diagnosis was a bacterial infection for which she started amoxicillin; however, subsequent radiography of the painful leg revealed suspicious lesions. The patient's admission labs revealed pancytopenia as shown below in Table 1.

**Table 1:** Complete blood count (CBC) upon admission revealed pancytopenia

Laboratory Test	Patient’s Laboratory Value	Normal Lab Range
Hemoglobin	6.0 g/dL	12-15 g/dL
Platelets	107x10^9^/L	150-450x10^9^/L
Leukocyte	1.29x10^9^/L	4.5-10.9x10^9^/L

**Figure 1:**
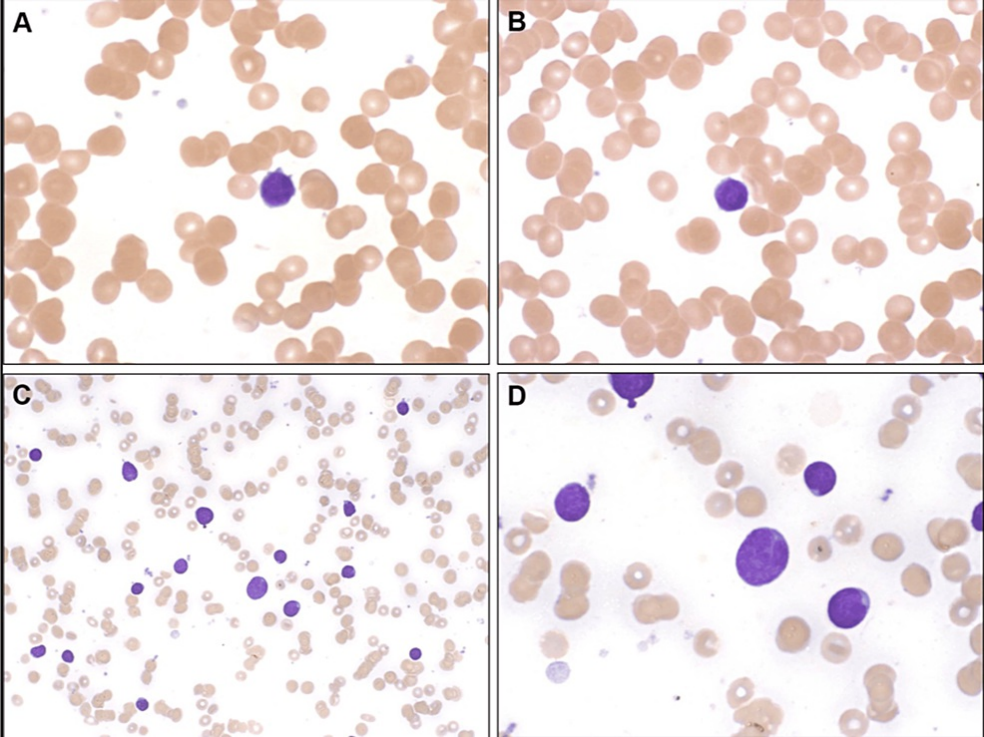
Peripheral smear prior to induction therapy demonstrated 9% blasts in the peripheral blood (A/B) and 39% phenotypically abnormal precursor B cells in the bone marrow (C/D) consistent with precursor B cell ALL Magnification of 20x (A/B/D) and 100x (C), respectively

A peripheral blood smear revealed 9% blasts (Figure 1 A/B). Bone marrow studies revealed 39% phenotypically abnormal B-precursor cells (Figure 1 C/D). A detailed cytogenetic profile did not reveal high or low-risk mutations.

She was diagnosed with standard risk B-precursor ALL and began induction therapy according to the COG protocol AALL0932 with intravenous cytarabine, vincristine, dexamethasone, pegaspargase, and intrathecal methotrexate. After 30 days of induction therapy, a repeat bone marrow biopsy revealed persistent disease with 1.5% blasts on flow cytometry. She was started on consolidation therapy with COG high-risk protocol AALL1131, including cyclophosphamide, cytarabine, mercaptopurine, intrathecal methotrexate, vincristine, and pegaspargase.

During the last week of consolidation therapy, she was admitted to the intensive care unit for septic shock with acute respiratory distress syndrome (ARDS), and she was found to have *Candida tropicalis* fungemia. Viral serologies, including Ebstein-Barr virus (EBV) and cytomegalovirus (CMV), were negative. She required mechanical ventilation for 58 days, and hospitalization was further complicated by disseminated HSV infection with splenic and hepatic microabscesses. No chemotherapy was administered during this hospitalization. On day 63 of hospitalization, a bone marrow examination demonstrated no residual disease on flow cytometry. Thereafter, weekly peripheral blood flow cytometric evaluations continued to show remission for nine months until she was discharged from the hospital following rehabilitation. Unfortunately, 51 weeks after the last chemotherapy dose and four months following discharge, she was found to have relapsed disease. She began standard four-drug re-induction therapy. Following re-induction, she unfortunately developed Escherichia (E.) coli septicemia and passed away.

## Discussion

There have been over 60 reported cases of transient SR in pre-B ALL and myelodysplasia followed by relapse of disease [1-4]. One limitation of comparison between these cases of SR is the technology used for identification. Dozens of SR cases were documented before advances such as complex cytogenetic analyses and flow cytometry; thus, uncertainty exists regarding the nature and mechanism of SR in these early documented cases. Many of these documented cases involve pre-B ALL with aplastic bone marrow undergoing SR to cellular normalization followed by overt leukemia with predictable spikes in immature lymphocytes [2,5]. There have been far fewer cases of SR following overt leukemia reflective of the natural history of the disease in the same patient rather than two distinctive remission processes [4].

Several mechanisms have been proposed to explain the phenomenon of SR, including endogenous or iatrogenic steroid effects, cytokine mediation following sepsis, and cytotoxic T-cell surveillance of cancer cells [6,7]. In leukemia specifically, the presumed immunologic mechanism of cytokine storm stems from the recent or concurrent history of febrile illness with bacterial or viral septicemia. Cytokines, including TNF-a, IFN-g, and IL-2, have been demonstrated to have anti-tumor effects in vitro [8]. It has been proposed that fungal toll-like receptors (e.g., TLR2/TLR9), especially when compounded by a cytokine storm, may shift the immune system into an activated tumor microenvironment stimulating antileukemic effects. T-cell surveillance has also been proposed as a mechanism of SR [9]. In one study, T cells were shown surrounding malignant B lymphoblasts on a marrow biopsy specimen [10]. The antineoplastic surveillance role of regulatory T cells and cytotoxic T cell-induced apoptosis is known. Additionally, studies have shown that leukemic cells often express MHC I/II in addition to co-stimulatory molecules, such as CD80/86, highlighting the susceptibility of these cells to T lymphocyte attack [11]. In our case, peripheral blood at the time of remission with less than 1% blasts showed that lymphocytes were primarily T-cells. Perhaps the mechanism of SR is multifactorial, with sepsis triggering the release of cytokines that up-regulate lymphocyte surveillance and destruction of cancer cells. B-cells and T-cells may recognize blasts, or antibodies formed against infectious agents, i.e. fungal antigens, which may cross-react with antigens on leukemic blasts leading to SR [11]. Finally, hormonal involvement has been proposed as a mechanism of SR, as profound increases in adrenocorticotropic hormone (ACTH) and cortisol are known to be released as a stress response to septicemia, and exogenous corticosteroids have been shown to aid in remission of leukemia [8]. In our case, dexamethasone was used during induction although it was discontinued at the onset of her acute deterioration.

The case we present is an example of SR following pre-B ALL in the current era of diagnostic adeptness. There have been several cases of SR following fungal infection; however, this is the first reported case of SR following *Candida tropicalis* sepsis in pediatric high-risk ALL. The phenomenon of SR as seen in our patient appeared to be transient in nature. Since 1996, less than 20 cases of SR in pediatric ALL have been reported in the literature, with each of them reporting a relapse of disease within weeks to months [12]. SR; however, has been reported more commonly in children with AML, and the interval range to relapse is 0-16 months with a mean of seven months [13]. A review of the literature regarding SR in ALL suggests a similar mean time to relapse although the size of the studies is smaller [2-4,7-10,12]. Given that all reported SR cases resulted in a relapse, which worsens the prognosis of ALL, we contemplate it would be favorable to implement low-dose maintenance chemotherapy following SR such as methotrexate or 6-mercaptopurine. There have been no cases to date treated with chemotherapy in the interval between SR and relapse. While no evidence-based protocols for the treatment of SR in ALL exist, we hope cases like this provide insight into the transiency of remission and the need for close follow-up.

Finally, treatment of ALL in the recent era has much improved with technological advances in immunotherapy, such as blinatumomab, which is now FDA-approved for minimal residual disease, or chimeric antigen receptor T-cell (CAR-T) therapy [14-15]. Universal CAR-T cells are FDA-approved for use in patients with ALL; however, antigen escape remains an issue and relapses have occurred [15].

## Conclusions

We report a rare case of *Candida tropicalis* sepsis in a pediatric patient with high-risk ALL who developed spontaneous remission of leukemia. Spontaneous remission of cancer is a poorly understood phenomenon reported sporadically throughout literature following infection, with a limited understanding of its physiologic basis. Unfortunately, all reported cases eventually have resulted in relapse; thus, future studies are needed to better understand the mechanism behind spontaneous remission as well as the best maintenance therapy intervention while in remission.

## References

[1] Cole WH, Everson TC (1956). Spontaneous regression of cancer: preliminary report. Ann Surg.

[2] Lynggaard LS, Marquart HV, Kjeldsen E, Madsen HO, Hasle H (2016). Acute lymphoblastic leukemia presenting with pancytopenia followed by a 14-month-long period of transient remission possibly supporting the adrenal hypothesis of leukemogenesis. J Pediatr Hematol Oncol.

[3] Purohit A, Aggarwal M, Kumar S (2015). Spontaneous remission of adult acute lymphoblastic leukemia: a very rare event. Indian J Hematol Blood Transfus.

[4] Höres T, Wendelin K, Schaefer-Eckart K (2018). Spontaneous remission of acute lymphoblastic leukemia: a case report. Oncol Lett.

[5] Puré E, Allison JP, Schreiber RD (2005). Breaking down the barriers to cancer immunotherapy. Nat Immunol.

[6] Iqbal A, Weinstein J, Angelova V, Dighe D, Giordano L (2018). A rare case of spontaneous remission of terminal deoxynucleotidyl transferase negative B-acute lymphoblastic leukemia. J Pediatr Hematol Oncol.

[7] Schmiegelow K, Vestergaard T, Nielsen SM, Hjalgrim H (2008). Etiology of common childhood acute lymphoblastic leukemia: the adrenal hypothesis. Leukemia.

[8] Kirtek T, Hamdan H, Van Arnam JS, Park S, Kovach AE, Pillai V, Weinberg OK (2023). Spontaneous remission of acute lymphoblastic leukemia: a series of nine cases and a review of literature. Int J Lab Hematol.

[9] Boonchalermvichian C, Xie Y, Brynes RK, Siddiqi IN (2012). Spontaneous, transient regression of B lymphoblastic leukemia in an adult patient: a variant presentation of prodromal/pre-ALL. Leuk Res.

[10] Yang D, Zhang X, Zhang X, Xu Y (2017). The progress and current status of immunotherapy in acute myeloid leukemia. Ann Hematol.

[11] Mitterbauer M, Fritzer-Szekeres M, Mitterbauer G (1996). Spontaneous remission of acute myeloid leukemia after infection and blood transfusion associated with hypergammaglobulinaemia. Ann Hematol.

[12] Matloub YH, Brunning RD, Arthur DC, Ramsay NK (1993). Severe aplastic anemia preceding acute lymphoblastic leukemia. Cancer.

[13] Paul R, Remes K, Lakkala T, Pelliniemi TT (1994). Spontaneous remission in acute myeloid leukaemia. Br J Haematol.

[14] Queudeville M, Ebinger M (2021). Blinatumomab in pediatric acute lymphoblastic leukemia-from salvage to first line therapy (a systematic review). J Clin Med.

[15] Pehlivan KC, Duncan BB, Lee DW (2018). CAR-T cell therapy for acute lymphoblastic leukemia: transforming the treatment of relapsed and refractory disease. Curr Hematol Malig Rep.

